# The ultra-processed foods paradox: When classification overlooks nutritional complexity

**DOI:** 10.1017/S1368980025101675

**Published:** 2026-02-04

**Authors:** Mina Babashahi, S Faezeh Hashemi Moghaddam

**Affiliations:** 1 Nutrition Research Center, Shiraz University of Medical Sciences, Shiraz, Iran; 2 Department of Community Nutrition, School of Nutrition and Food Sciences, Shiraz University of Medical Sciences, Shiraz, Iran; 3 Department of Clinical Nutrition, School of Nutrition and Food Sciences, Shiraz University of Medical Sciences, Shiraz, Iran

There are some systems like NOVA and SIGA that categorise foods into groups with different levels of processing where one such group includes ultra-processed foods (UPF). Here, we want to express several concerns with these systems emphasising NOVA as most of the research focuses on it. NOVA, developed by Monteiro et al at the University of São Paulo in Brazil, is a food system categorising foods into 4 groups based on extent of processing: (1) unprocessed foods, (2) processed culinary ingredients, (3) processed foods and (4) UPF^(1,2)^. This system has gained widespread attention from nutritionists, the media and consumers. Although some studies have shown sleep disturbances, increased inflammatory markers, all-cause mortality and umbrella reviews have shown an association between high UPF consumption with increased risk of a variety of chronic conditions such as obesity, type 2 diabetes mellitus, hypertension, impaired renal function, colorectal cancer, Crohn’s disease, sleep disturbance, functional constipation, binge eating disorders, irritable bowel syndrome, asthenozoospermia, functional dyspepsia, dementia and all-cause mortality,^(3–7)^ there are some gaps and limitations that warrant consideration in the system and its categorisation.

A major methodological limitation of the NOVA classification is the heterogeneity within the UPF (group 4) category, which groups together products with vastly different nutritional profiles and potential health impacts. Therefore, it is possible that the results of some current research based on this system encourage avoiding consuming UPF regardless of whether some of these foods are potentially beneficial for health. All UPF are placed in one category and treated as equally bad for health. In this regard, fortified foods are considered UPF according to this system. Fortification of wheat flour, salt, dairy products, grains, and even juices and cakes with micronutrients such as iron, folic acid, zinc, vitamin D, B-complex vitamins, iodine and fibre can improve health of individuals and prevent micronutrient deficiencies, anaemia and serious diseases like neural tube defect in infants, hypothyroidism and goitre, prevent conditions like constipation and compensate for some of the nutrient losses during the processing of foods like losses of B-complex vitamins during the removal of bran in bread and rice.

Formulas including infant formulas and medical nutrition products (e.g. enteral nutrition used in intensive care units) are considered UPF. While formulas are undeniably highly processed, they are necessary for babies who cannot be breastfed due to the medical conditions or insufficient milk production of their mothers or for babies with metabolic diseases such as phenylketonuria. Similarly, hospital formulas are often life-saving for patients with cancer, anorexia nervosa or those who can’t meet their increased needs of calories and nutrients and cannot consume adequate food orally. One of the issues with these classifications is that they do not consider these therapeutic contexts and positive health effects of some UPF.

While many studies based on this system emphasise the effects of UPF on health, they fail to consider potential environmental benefits associated with certain UPF products. For instance, although conventional meat production and consumption negatively impact climate change, water resources and economic sustainability, plant-based meat alternatives offer significant advantages. Beyond substantially reducing greenhouse gas emissions and water usage, these products can also provide nutritious options. Notably, in high-income countries, replacing some meat consumption with plant-based alternatives can yield more health benefits, while in low-income settings, such alternatives can enhance access to affordable nutrition where animal products may be economically prohibitive^(8)^.

These systems call out additives as a hallmark of processed foods and treat them all equally, while artificial colours and flavours have different health effects from natural forms. Actually, many food additives like emulsifiers, stabilisers and some preservatives are not considered to increase risk of diseases, and they are necessary for some kinds of foods like some dairy products to maintain their quality, safety and shelf-life.

Interest in foods and beverages low in energy, sugar, fat or salt has been increasing. While they are healthier choices and are good for people with some conditions like obesity and diabetes, they are still categorised as UPF and less healthy foods according to the food classification systems.

The NOVA system considers industrialised foods such as soft drinks, packaged snacks and instant noodles as UPF. It is commonly assumed that home-cooked meals are healthier than industrialised products, but it’s not always true. Some home-made foods like French fries, fried foods, home-made breads and the foods that need deep frying, high-temperature baking or processed ingredients can be as low nutritional value as some UPF because of acrylamide, trans fats and some other carcinogenic substances that are produced during the process of cooking, whether in the home or by a manufacturer. Furthermore, the health problems such as overweight, obesity, type 2 diabetes and CVDs associated with excessive consumption of added sugar, salt and oil originate not only from UPF but also from home-cooked meals. Thus, these problems cannot be viewed as disadvantages unique to UPF.

While conventional food classification systems, such as SIGA, often assign a low health grade to canned foods due to their nutritional profile (e.g. high salt) and ultra-processed nature, a strictly nutrient and processed-based assessment fails to account for critical contextual aspects. In situations of geopolitical instability, natural disasters, war or famine, the same UPF packaged or canned foods become vital lifelines because of their energy density, safety and long shelf-life. So, to make sure these systems are both academically rigorous and practically applicable, scientific research must develop credible mechanisms to contextually adjust the health valuation of such foods during crisis situations, noting their crucial role in ensuring food security and survival.

Also, despite the increase of studies showing the impact of UPF on health, most of them haven’t used validated tools or FFQ based on NOVA or other systems^(5,6)^. Two FFQ are validated based on NOVA, but neither of these was used for the assessment of UPF consumption in research^(9,10)^. It means that the results drawn from many studies may not be valid, as self-reported dietary intake can be inaccurate and biased.

## Conclusion

In summary, while the current food processing classification systems provide valuable information about food processing, they have some limitations and paying attention to these points about these systems can be important to prevent overestimation or underestimation of the risk factors affecting health. The current classification of UPF fails to discriminate between nutrient-dense and nutrient-poor UPF, which is a critical shortcoming for developing accurate and precise studies and dietary guidelines. We propose either a substantial revision of the existing framework or the development of a novel classification system that integrates multiple dimensions of food processing, accounts for varying contexts of food security and is grounded in causal evidence from randomised controlled trials and longitudinal studies.
